# Lifestyles and socio-cultural factors among children aged 6–8 years from five Italian towns: the MAPEC_LIFE study cohort

**DOI:** 10.1186/s12889-017-4142-x

**Published:** 2017-03-07

**Authors:** Francesco Bagordo, Antonella De Donno, Tiziana Grassi, Marcello Guido, Gabriele Devoti, Elisabetta Ceretti, Claudia Zani, Donatella Feretti, Milena Villarini, Massimo Moretti, Tania Salvatori, Annalaura Carducci, Marco Verani, Beatrice Casini, Sara Bonetta, Elisabetta Carraro, Tiziana Schilirò, Silvia Bonizzoni, Alberto Bonetti, Umberto Gelatti, MAPEC_LIFE study group, Francesca Serio, Mattia De Giorgi, Adele Idolo, Tiziano Verri, Loredana Covolo, Francesco Donato, Andrea Festa, Rosa Maria Limina, Ilaria Zerbini, Cristina Fatigoni, Sara Levorato, Silvano Monarca, Samuele Vannini, Gabriele Donzelli, Beatrice Bruni, Giacomo Palomba, Silvia Bonetta, Marta Gea, Giorgio Gilli, Cristina Pignata, Valeria Romanazzi, Camilla Furia, Roberta Codenotti, Paolo Colombi, Stefano Crottini, Laura Gaffurini, Licia Zagni

**Affiliations:** 1 Department of Biological and Environmental Science and Technology, University of Salento, Via Prov.le Lecce-Monteroni, 73100 Lecce, Italy; 2 Department of Medical and Surgical Specialties, Radiological Sciences and Public Health, University of Brescia, Brescia, Italy; 3 Department of Pharmaceutical Sciences, University of Perugia, Perugia, Italy; 4 Department of Biology, University of Pisa, Pisa, Italy; 5 Department of Translational Research, N.T.M.S., University of Pisa, Pisa, Italy; 6 Department of Public Health and Pediatrics, University of Torino, Torino, Italy; 7 Comune di Brescia, Brescia, Italy; 8 Centro Servizi Multisettoriale e Tecnologico - CSMT Gestione S.c.a.r.l., Brescia, Italy

**Keywords:** Children, Lifestyles, Exposure, Air pollution, Questionnaire, MAPEC_LIFE Study

## Abstract

**Background:**

Lifestyles profoundly determine the quality of an individual’s health and life since his childhood. Many diseases in adulthood are avoidable if health-risk behaviors are identified and improved at an early stage of life. The aim of the present research was to characterize a cohort of children aged 6–8 years selected in order to perform an epidemiological molecular study (the MAPEC_LIFE study), investigate lifestyles of the children that could have effect on their health status, and assess possible association between lifestyles and socio-cultural factors.

**Methods:**

A questionnaire composed of 148 questions was administered in two different seasons to parents of children attending 18 primary schools in five Italian cities (Torino, Brescia, Pisa, Perugia and Lecce) to obtain information regarding the criteria for exclusion from the study, demographic, anthropometric and health information on the children, as well as some aspects on their lifestyles and parental characteristics. The results were analyzed in order to assess the frequency of specific conditions among the different seasons and cities and the association between lifestyles and socio-economic factors.

**Results:**

The final cohort was composed of 1,164 children (50.9 boys, 95.4% born in Italy). Frequency of some factors appeared different in terms of the survey season (physical activity in the open air, the ways of cooking certain foods) and among the various cities (parents’ level of education and rate of employment, sport, traffic near the home, type of heating, exposure to passive smoking, ways of cooking certain foods). Exposure to passive smoking and cooking fumes, obesity, residence in areas with heavy traffic, frequency of outdoor play and consumption of barbecued and fried foods were higher among children living in families with low educational and/or occupational level while children doing sports and consuming toasted bread were more frequent in families with high socio-economic level.

**Conclusions:**

The socio-economic level seems to affect the lifestyles of children enrolled in the study including those that could cause health effects. Many factors are linked to the geographical area and may depend on environmental, cultural and social aspects of the city of residence.

## Background

According to the World Health Organization definition, lifestyles are ways “of living based on identifiable patterns of behaviour which are determined by the interplay between an individual’s personal characteristics, social interactions, and socioeconomic and environmental living conditions” [1]. They represent a group of factors that profoundly determine the quality of an individual’s health and life [2].

Lifestyles include living conditions editable since they are determined by the choices made by each individual in the course of its existence [3] and can be divided into health-risk behaviors and health-promoting behaviors. Health-risk behaviors (e.g., smoking habit, alcohol use, sedentary lifestyle, high-calorie diet, living in polluted areas) are activities or conditions that increase a person’s vulnerability or susceptibility to negative health outcomes (cancer, obesity, diabetes, cardiovascular diseases) [4]. In contrast, health-promoting behaviors (physical activity, proper nutrition, etc.) entail an increase in the psycho-physical well-being which prevent morbidity and premature mortality [5–7]. The socio-economic context, namely that resulting from the interaction of several factors such as culture, education, income, family structure, home and work environment, can affect many behavioral and decision-making aspects playing a role on health literacy, or on the availability of goods and services [8].

Life quality and health in adulthood often depends on the living habits adopted since early years. Many studies revealed that health-risk beaviours in childhood may have short-term consequences [9] and could be carried into adulthood determining also long-term consequences [9, 10], as well as being a substantial economic burden [11–13]. Therefore, many diseases in adulthood are avoidable if health-risk behaviors are identified and improved at an early stage of life [7].

The purpose of the present research was to a) characterize a cohort of children aged 6–8 years selected in order to perform an epidemiological molecular study in five Italian cities (the MAPEC_LIFE - Monitoring Air Pollution Effects on Children for Supporting Public Health Policy - study), b) investigate lifestyles of the children that could have effect on their health status, and c) assess possible association between lifestyles and socio-cultural factors.

## Methods

### Study design

This study was included in the MAPEC_LIFE project (LIFE12 ENV/IT/000614) [14], a multicenter cohort study funded by the European Union’s LIFE+ Programme which aims to assess the association between concentrations of monitored atmospheric pollutants and early biological effects on children aged 6–8 years living in areas with varying levels of air pollution and to build a model for estimating global genotoxic risk that can be used to support public health policy.

In order to accurately correlate biological effects with the concentration of atmospheric pollutants, the parents of children participating in the MAPEC_LIFE study were asked in two different seasons to fill in a pre-validated questionnaire [15] to assess lifestyles and exposure factors linked to the home context that could have a confounding effect on the measured responses.

### Cities involved in the study

**Fig. 1:**
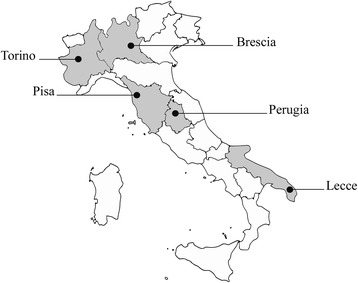
Localization of the five cities involved in the MAPEC_LIFE study

The study was conducted in five Italian cities (Fig. 1), differing in geographical, environmental, demographic and socio-economic terms.

Torino (TO) (890,529 inhabitants [16]) is situated in the north-west of the Pianura Padana, the Plain of the river Po, in a metropolitan area with more than two million inhabitants which is one of the most important economic and industrial sites in Italy [17]. The city’s core and the surrounding urban area pay the price of economic development in terms of exposure to high levels of air pollution [18, 19]. Torino is the coldest of the cities included in the study [20], with an average annual temperature in the last 10 years of 13.2 °C (Torino-Caselle Weather Station).

Brescia (BS) (about 200,000 inhabitants) is part of an extensive metropolitan area, highly industrialized, with a high level of motor vehicle traffic. It is situated in the high Pianura Padana, one of the most highly polluted areas in Europe [21, 22] and has a humid subtropical climate [23]. It is characterized by one of the highest rates of immigration in Lombardia and the highest number of foreign residents. A recent study [24] includes it with Pisa among cities characterized by high levels of economic development, well-being and cultural activity.

Pisa (PI), with just under 90,000 inhabitants, is the smallest of the cities in the study. It lies a few kilometers from the mouth of the river Arno in a flat area that hosts a large industrial estate. The climate is affected by the vicinity of the sea, which tends to mitigate both the rigors of winter and the heat of summer. The social fabric is influenced by a large number of foreign residents and the presence of many university students.

Perugia (PG) (about 166,000 inhabitants) is in central Italy at an altitude of more than 400 m above the sea level. It has a temperate climate with an average annual temperature in the last decade of just over 14 °C. The economy is mainly based on the tertiary sector while the industrial sector is composed above all of textiles and food processing. In PG too, a large part of the population is accounted for by foreigners and students, although the old age index (percentage ratio between population aged 65 years and over and population aged 0–14 years) is much higher (163.5) than the national average.

Lecce (LE) has just over 90,000 inhabitants and is situated in south-east Italy, in the center of the Salento peninsula. It has a Mediterranean climate, with mild winters and hot humid summers. Like PE it is a city with a solid but largely static economy [24], based essentially on food processing, handicrafts, tourism and services, and is characterized by the tendency towards an ageing population.

### Recruitment of the children

Recruitment was conducted randomly among the children attending first, second and third year classes of 18 primary schools located in the five cities of the study (selected in order to recruit at least 200 children from at least three schools per city, located in the urban area and in different neighborhoods, far from point sources of contamination, and free from radioactive indoor pollution) in accordance with a protocol [14] shared by the Research Units of the Universities of Brescia, Salento (LE), Perugia, Pisa and Torino. Participation in the study was on a voluntary basis after inviting the parents of all children attending these classes by distribution of a study parcel which contained a) a fact sheet informing the children’s parents about the study, its objectives and methods and; b) the informed consent form. The parents who had agreed to participate in the study gave the approval to the recruitment of their children by signing the consent form.

### Questionnaire administration

The parents of children participating in the study were asked to fill in a previously validated questionnaire [15], composed of 148 questions, subdivided into various sections: criteria for exclusion from the study (age below 6 years or equal/above nine, residence in cities other than those involved in the study, the presence of serious illness, exposure to radiotherapy or chemotherapy in the 12 months preceding the investigation, exposure to radiographic testing in the month preceding the investigation, use of dental braces); the child’s personal information (gender, date and nation of birth); the child’s weight and height; information on the child’s health status (respiratory problems beyond the common cold and consumption of medicines in addition to common remedies such as antibiotics, antipyretics and anti-inflammatory agents); domestic environment (intensity of traffic near the home, including heavy goods vehicles, fuel used for heating and cooking, presence of gas boilers, stoves and fireplaces inside the dwelling, presence of smokers inside the dwelling, use of solvents for hobbies, i.e., paint thinner, white spirit, acetone, turpentine, toluene, etc.); information on the child’s lifestyle (exercise, consumption of dishes cooked in certain ways); parents’ characteristics (nation of birth, level of education, occupation, smoking habits); information on the child’s eating habits. All the questions were of the “closed answer” type.

The parents of each child filled in the questionnaire twice in two different seasons (Season I, winter 2014–2015, and Season II, late spring 2015), directly on a specially created web platform or in paper format. In the latter case the completed questionnaires were returned to the teachers and uploaded to the web platform by the personnel of the various Research Units. The software supporting the web platform assigned an alphanumeric identification code to each questionnaire and related data were transferred automatically to spreadsheets for processing.

### Sample size

The final cohort was composed of all children whose parents completed valid questionnaires in both seasons. In total, 1164 children, 3.1% of the total children aged 6–8 years living in the cities involved in the study, were enrolled.

### Data processing

The exact age at testing was measured by calculating the time interval between the date of birth given on the questionnaire and the date of filling the questionnaire. The data for weight and height given by the parents were used to calculate the children’s body mass index (BMI) (weight [kg]/height [m] squared), which was used in turn to assess whether the child was underweight (UW), of normal weight (NW), overweight (OW) or obese (OB). In accordance with the indications of the International Obesity Task Force (IOTF), OW and OB were defined with reference to the BMI threshold values for boys and girls aged 2–18 years, calculated by Cole et al. [25] on the basis of adult values (overweight = 25 kg/m^2^; obesity = 30 kg/m^2^). The cut-off for the UW category was set at the “-2 z-score” on the basis of the BMI threshold values set out in Cole et al. [26]. The data thus obtained were statistically processed using MedCalc Software version 12.3 (MedCalc Software bvba, Ostend, Belgium).

The answers in the questionnaires were analyzed in order to assess the average values (± standard deviation) of quantitative variables (i.e., age, height, weight, BMI, use of stoves and fireplaces), and the frequency of children with specific characteristics in the two seasons and the five cities participating in the study. Quantitative data were compared by one-way ANOVA, and frequencies by the chi-squared test. Differences were considered significant at *p < 0.05*.

In addition, the association between certain behavioral factors (e.g., exposure to passive smoking, living in areas with heavy traffic, obesity, physical activity, consumption of foods cooked by risky methods), and socio-economic level of the children’s families was assessed by chi-squared test taking into account the overall level of parents’ education and father’s employment. The parents’ level of education was calculated as total years of schooling, depending on the mother’s and father’s educational level reported in the questionnaire. According to the Italian school system, we assigned 0 years when parents reported not having any degree; 5 years to primary school certificate; 3 years to junior high school diploma; 5 years to high school diploma; and 5 years to graduate. The employment level of the father was divided into four categories: level I (businessmen, managers, professionals); level II (office worker); level III (manual worker, craftsman); level IV (unemployed).

### Ethical aspects

The study was approved by the competent Ethical Committees in each involved city. All the data were gathered and analyzed in accordance with Italian Legislative Decree 196 of 30/6/2003 (“protection of personal data”) and subsequent additions, for the purposes of research.

## Results

### Recruitment

The results of the recruitment and questionnaire administration activities are illustrated in Table 1. A total of 3,144 parcels were distributed to parents of children attending first, second and third year classes in selected schools in the five towns; 1,767 (56.2%) correctly compiled consent forms were gathered and the same number of questionnaires were given during the first season to the parents who decided to participate. Overall, 1,356 questionnaires (76.7%) were valid, after excluding those that were either incomplete (*n* = 265) or did not meet the criteria for inclusion (*n* = 146).

**Table 1:** Results of recruitment and questionnaire administration activities

	BS	LE	PG	PI	TO	TOTAL
Selected schools (n)	4	3	4	4	3	18
Distributed parcels (n)	600	750	711	527	556	3144
Season I						
Informed consents/distributed questionnaires (n)	406	343	416	282	320	1767
Completed questionnaires (n)	325	284	350	268	275	1502
Valid questionnaires (n)	289	270	296	246	255	1356
Season II						
Distributed questionnaires (n)	283	266	276	242	251	1318
Completed questionnaires (n)	265	249	249	219	237	1219
Valid questionnaires (n)^a^	250	242	235	210	227	1164

^a^final cohort

In the second season 1,318 questionnaires were handed in. Of these, 1,164 (final cohort) were found to be valid, with an average loss with respect to the first season of 14.2%, ranging from 10.4% in LE to 20.6% in PG.

### Characteristics of the recruited children

Children included in the final cohort (Table 2) were 50.9% boys and 95.4% born in Italy. Their mean age at the time of recruitment was 7.34 ± 0.87 years while the average BMI was 16.5 ± 2.6 kg/m^2^. Regarding their weight status large number of children were overweight (12.5% in the Season I and 13.7% in Season II) or obese (16.2–14.8%). No significant difference between the two surveys was recorded, although during the second season there was a significantly diverse distribution (*p < 0.05*) of normal-weight children among the various cities, with the highest frequency seen in BS (75.6%), followed by TO (69.6%), PG (68.9%), LE (64.5%) and PI (62.9%).

**Table 2:** Demographic and anthropometric variables, health status and physical activity among children participating in the study

	BS	LE	PG	PI	TO	MAPEC_LIFE cohort	*P-*value
Males (%)	47.2	49.2	58.3	46.2	53.3	50.9	*0.057* ^a^
Born in Italy (%)	94.4	97.9	96.2	93.8	94.7	95.4	*0.204* ^a^
Age (years)							
*Season I*	7.42	7.38	7.36	7.15	7.33	7.34	*0.051* ^b^
*Season II*	7.76	7.53	7.66	7.53	7.67	7.63	*0.052* ^b^
Height (cm)							
*Season I*	124.8	125.4	125.1	123.6	125.0	124.8	*0.183* ^b^
*Season II*	127.1	126.7	126.8	126.1	126.6	126.7	*0.756* ^b^
Weight (kg)							
*Season I*	25.5	26.4	26.5	25.5	25.7	25.9	*0.102* ^b^
*Season II*	26.5	26.9	27.1	26.7	26.5	26.7	*0.725* ^b^
BMI (kg/m^b^)							
*Season I*	16.3	16.7	16.8	16.6	16.3	16.5	*0.076* ^b^
*Season II*	16.2	16.6	16.7	16.7	16.5	16.6	*0.200* ^b^
UW (%)^c^							
*Season I*	3.6	4.1	3.8	2.9	2.2	3.4	*0.781* ^a^
*Season II*	2.4	4.5	1.3	3.3	3.5	3.0	*0.290* ^a^
NW (%)							
*Season I*	70.8	66.9	66.0	63.3	72.2	68.0	*0.244* ^a^
*Season II*	75.6	64.5	68.9	62.9	69.6	68.5	*0.028* ^a^
OW (%)^d^							
*Season I*	13.2	13.2	11.9	15.7	8.8	12.5	*0.278* ^a^
*Season II*	10.4	15.7	14.0	16.2	12.8	13.7	*0.355* ^a^
OB (%)^e^							
*Season I*	12.4	15.7	18.3	18.1	16.7	16.2	*0.396* ^a^
*Season II*	11.6	15.3	15.7	17.6	14.1	14.8	*0.454* ^a^
Respiratory problems (%)^f^							
*Season I*	17.2	15.7	16.2	15.7	18.1	16.6	*0.951* ^a^
*Season II*	19.2	14.5	14.9	15.7	15.4	16.0	*0.624* ^a^
Consumption of medicines (%)^g^							
*Season I*	5.6	3.3	7.7	4.8	4.0	5.1	*0.231* ^a^
*Season II*	4.8	5.4	6.0	4.8	4.8	5.2	*0.973* ^a^
Sport (equal or above 3 times/week) (%)							
*Season I*	42.0	57.0	44.7	42.4	37.4	44.8	*<0.001* ^a^
*Season II*	44.8	57.9	47.7	47.6	40.1	47.7	*0.003* ^a^
Outdoor sports (%)							
*Season I*	31.6	26.4	31.9	27.1	23.8	28.3	*0.224* ^a^
*Season II*	41.6*	35.5*	40.9*	36.7*	28.2	36.7*	*0.021* ^a^
Swimming (indoor) (%)							
*Season I*	3.6	13.6	3.4	5.7	3.5	6.0	*<0.001* ^a^
*Season II*	5.2	12.0	8.5	5.7	4.4	7.2	*0.008* ^a^
Outdoor play (above 1 h/day) (%)							
*Season I*	35.6	25.6	28.5	40.0	36.1	33.0	*0.003* ^a^
*Season II*	72.0*	59.9*	66.4*	80.0*	74.4*	70.3*	*<0.001* ^a^

^a^significance level by chi-squared test among frequencies in the various cities

^b^significance level by one-way ANOVA among average values in the various cities

^c^BMI cut-off point for age and sex set at “-2z score” according to Cole et al. [26]

^d^Include children whose BMI is comprised between the 85th and 95th percentiles specific for age and sex according to Cole et al. [25]

^e^Include children whose BMI is above the 95th percentile specific for age and sex according to Cole et al. [25]

^f^beyond the common cold

^g^in addition to common remedies such as antibiotics, antipyretics and anti-inflammatory agents

*p-value <0.05 by chi-squared test between frequencies in the two seasons

**Fig. 2:**
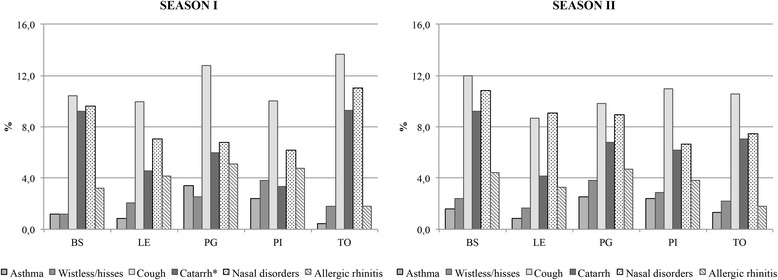
Frequency of main respiratory disorders in the two seasons. Frequency of main respiratory disorders that occurred in children in the month preceding the survey carried out in the season I and season II. * *p-*value <0.05 by chi-squared test between frequencies in the two seasons

16.6% of the children in season I and 16.0% in season II suffered from respiratory problems beyond the common cold with significant differences between the cities regarding the incidence of catarrh (Fig. 2) in winter, the highest values in TO (9.3%) and BS (9.2%) and the lowest in LE (4.5%) and PI (3.3%). The average rate of use of medicines in addition to common remedies such as antibiotics, antipyretics and anti-inflammatory agents in the 6 months before sampling was 5.1% in season I and 5.2% in season II.

Concerning physical activity, 44.8% of the children did sport three or more times a week in season I and 47.7% in season II. The percentages of children doing sport in the open air increased significantly (*p < 0.05*) from the first season (28.3%) to the second season (36.7%), as did the percentages of children playing outdoors for more than 1 h a day (33.0% in the first season, 70.3% in the second). Significantly different frequencies among the cities were recorded.

### Characteristics of the parents

The parents of the recruited children exhibited significantly different characteristics (*p < 0.05*) in terms of gender and city (Table 3). 84.7% of the mothers and 88.1% of the fathers were born in Italy with the highest values seen in LE (92.4% of mothers and 95.7% of fathers) and the lowest in PI (77.4% of mothers and 82.9% of fathers). The level of education is highest among the parents of PG, where 91.1% of mothers and 85.0% of fathers have at least a high-school leaving certificate (in BS only 78.4% of mothers and 68.8% of fathers). Overall, the mothers were more likely than the fathers to have this level of education (83.7 vs. 76.2%). In contrast, the rate of employment is higher among the fathers (73.3 vs. 89.2%). In addition, the fathers are more likely to be smokers (27.5%) than the mothers (19.2%), while TO had the highest frequency of smoking parents (36.6% of fathers and 26.0% of mothers) and PG the lowest (20.6% of fathers and 13.2% of mothers).

**Table 3:** Prevalence of variables regarding parents of children included in the MAPEC_LIFE cohort

	BS	LE	PG	PI	TO	mean	*P-*value^a^
Born in Italy (%)							
Mother	83.4	92.4	87.2	77.4	82.2	84.7	*<0.001*
Father	86.6	95.7	90.6	82.9	84.1	88.1	*<0.001*
High-school education or greater (%)							
Mother	78.4	85.1	91.1	85.0	79.3	83.7	*<0,001*
Father	68.8	78.2	85.0	72.8	76.4	76.2	*<0,001*
Employment rate (%)							
Mother	76.0	61.6	77.4	74.6	77.5	73.3	*<0.001*
Father	93.9	83.8	91.8	86.0	90.1	89.2	*0.002*
Smoking habits (%)							
Mother	16.6	20.2	13.2	20.5	26.0	19.2	*<0.001*
Father	22.1	28.6	20.6	30.4	36.6	27.5	*<0.001*
Smoking parents (%)							
Both	8.7	12.0	6.5	11.6	20.5	11.8	*<0.001*
One only	21.4	24.9	20.9	28.0	21.6	23.3	*0.058*
Neither	69.9	63.1	72.7	60.4	57.9	65.0	*<0.001*

^a^significance level by chi-squared test among frequencies in the various cities

### Domestic environment

The type of fuel used for domestic heating varies significantly among the various cities (Table 4). In BS and TO, the presence of district heating model is significant, while in the other cities natural gas is prevalent. Stoves are present in 7.3% of the dwellings. In most cases they run on wood or pellets (3.8%) and to a lesser extent on fossil fuels (2.4%) or electricity (1.1%). Open hearths are found in 23.7% of the dwellings, with a greater frequency in LE (37.2%) and a lower frequency in TO (7.0%). In the latter city, there is a greater frequency of gas boilers installed inside the apartment (18.5%). The main fuel used for cooking is natural gas (methane) in all the cities involved in the study. However, significant differences were found regarding the use of electricity, which is most frequent in BS (21.1%).

**Table 4:** Prevalence of different systems for heating and cooking food in the dwellings of enrolled children

	BS	LE	PG	PI	TO	mean	*P-*value^a^
Fuel used for heating (%)							
Wood/Pellet	6.9	7.2	12.4	1.4	1.5	6.2	*<0.001*
Diesel/Kerosene	0.6	1.4	5.1	2.6	10.1	3.9	*<0.001*
Gas	30.2	83.5	78.3	92.4	57.7	67.7	*<0.001*
Electricity	3.3	8.0	2.7	2.9	0.9	3.6	*<0.001*
District heating	59.1	0	1.1	0	29.8	18.5	*<0.001*
Stoves (%)							
Wood/Pellet	6.0	2.5	7.2	2.4	0.4	3.8	*<0.001*
Gas/kerosene	1.2	5.0	1.3	3.3	1.3	2.4	*0.220*
Electricity	0.4	2.1	1.3	1.0	0.9	1.1	*0.500*
Fireplaces (%)	23.6	37.2	34.9	13.8	7.0	23.7	*<0.001*
Gas boiler (%)	11.2	3.3	8.9	17.1	18.5	11.6	*<0.001*
Fuel used for cooking (%)							
Electricity/Induction	21.1	13.6	8.0	9.7	5.4	11.7	*<0.001*
Gas	78.2	85.7	89.2	90.3	94.6	87.4	*<0.001*
Wood/Charcoal	0.8	0.8	2.8	0	0	0.9	*0.005*

^a^significance level by chi-squared test among frequencies in the various cities

As shown in Table 5, TO had the highest percentage of children resident in areas judged to be of high traffic intensity by parents in both the first (53.7%) and second (60.4%) seasons. TO also had the largest number of children exposed to heavy vehicle traffic (17.6% in both seasons). Wood-burning stoves are most frequently used in the homes of PG (15.3 days/month in the first season and 13.3 days/month in the second season). Open-hearth fires were used most frequently in LE in the first season (7.3 days/month) and in TO in the second season (3.3 days/month). The habit of smoking in the home by one or more persons was recorded in 13.3% of cases in the first season and 13.5% in the second, while the presence of the child in closed rooms with smokers was declared by 3.5% of parents in the first season and 2.8% in the second. The presence of the child in the kitchen during food cooking was recorded in 17.1% of cases during the first season and 14.3% during the second. The practice of cooking food on the griddle increased significantly (*p < 0.05*) between the first (56.5%) and second (61.2%) seasons, with the highest frequency seen in BS (62.4%) in season I and PI (69.5%) in season II. Lastly, the use of solvents for hobbies was seen in 4.0 and 3.3% of homes during the first and second seasons respectively, without appreciable differences between the cities or between survey periods.

**Table 5:** Prevalence of exposure factors linked to the home context in the study cohort

	BS	LE	PG	PI	TO	mean	*P-*value
Residence in areas with heavy traffic (%)							
*Season I*	35.6	24.8	33.6	22.9	53.7	34.2	*<0.001* ^a^
*Season II*	42.4	29.3	36.2	29.0	60.4	39.5	*<0.001* ^a^
Residence in areas with heavy traffic of heavy vehicles (%)							
*Season I*	14.8	7.0	6.0	6.2	17.6	10.4	*<0.001* ^a^
*Season II*	11.6	5.4	7.7	5.2	17.6	9.5	*<0.001* ^a^
Use of stoves (d/m)							
*Season I*	8.2	15.3	15.3	7.0	6.5	11.7	*0.052* ^b^
*Season II*	11.7	7.1	13.3	3.0	7.8	9.2	*0.081* ^b^
Use of fireplaces (d/m)							
*Season I*	3.1	7.3	6.0	2.3	1.8	5.2	*0.040* ^b^
*Season II*	1.3	3.1	2.3	0.6	3.3	2.2	*0.221* ^b^
Presence of smokers inside the dwelling (%)							
*Season I*	12.8	14.0	11.9	13.8	12.3	13.3	*0.952* ^a^
*Season II*	12.0	12.8	11.9	19.0	12.3	13.5	*0.142* ^a^
Staying in closed rooms with smokers (%)							
*Season I*	4.0	3.7	3.0	3.3	3.5	3.5	*0.981* ^a^
*Season II*	2.4	3.3	3.0	3.8	1.8	2.8	*0.725* ^a^
Frequent presence of the child in the kitchen during food cooking (%)							
*Season I*	19.2	14.0	14.5	17.6	20.3	17.1	*0.271* ^a^
*Season II*	13.2	16.5	10.6	12.9	18.5	14.3	*0.116* ^a^
Cooking on the griddle/barbecue (%)							
*Season I*	62.4	56.2	56.2	59.5	48.0	56.5	*0.027* ^a^
*Season II*	64.8	63.6	57.9	69.5*	50.2	61.2*	*<0.001* ^a^
Use of solvents for hobbies (%)							
*Season I*	2.0	4.5	4.7	5.7	3.1	4.0	*0.264* ^a^
*Season II*	1.6	3.7	3.4	3.3	4.4	3.3	*0.508* ^a^

^a^significance level by chi-squared test among frequencies in the various cities

^b^significance level by one-way ANOVA among average values in the various cities

*p-value <0.05 by chi-squared test between frequencies in the two seasons

The frequency of consumption of certain foods subject to particular cooking methods in the month before the survey was conducted (Table 6) varies between the two seasons and/or the five cities. Specifically, the consumption of foods cooked on the barbecue increased significantly between the first (30.1%) and second (38.9%) seasons, with the highest frequencies seen in PG (39.6%) in winter and PI (47.1%) in spring. In contrast, the children of TO had the lowest consumption in both sampling periods (16.3% in season I and 20.3% in season II). Interestingly, the frequency of consumption of foods prepared with this method of cooking is overall higher among children with parents born outside of Italy (40.6% in season I and 55.4% in season II) (not reported in table). In addition, significantly (*p* < 0.05) more frequent consumption of foods cooked on the griddle was recorded in BS in both the first (71.1%) and second (67.7%) seasons, of fried food in LE in the first season (81.4%), of toasted bread in PG in the first season (58.3%) and smoked foods in BS in the second season (23.6%). Finally, frequency of consumption of pizza cooked in a wood oven was found significantly different (*p* < 0.05) between the two season in BS (61.2% in season I and 69.6% in season II) and PI (60.5% in season I and 68.1% in season II).

**Table 6:** Children who consumed foods subject to risky cooking methods in the month before the survey

	BS	LE	PG	PI	TO	mean	*P-*value^a^
Barbecued foods (wood/charcoal) (%)							
*Season I*	25.3	37.2	39.6	31.9	16.3	30.1	*<0.001*
*Season II*	40.4*	44.6*	42.1	47.1*	20.3	38.9*	*<0.001*
Foods cooked on the griddle (%)							
*Season I*	71.1	58.7	54.0	58.1	57.3	60.0	*0.002*
*Season II*	67.6	60.7	54.9	66.7	61.2	62.2	*0.032*
Fried foods (%)							
*Season I*	75.5	81.4	77.0	69.0	80.6	76.9	*0.017*
*Season II*	72.8	82.2	77.4	78.1*	79.7	78.0	*0.141*
Toasted bread (%)							
*Season I*	56.2	45.5	58.3	49.0	48.5	51.6	*0.022*
*Season II*	54.0	50.4	53.3	50.0	46.7	51.4	*0.356*
Smoked foods (%)							
*Season I*	20.5	12.0	12.8	14.8	15.0	15.0	*0.075*
*Season II*	23.6	19.8	12.8	18.6	16.3	18.3	*0.033*
Pizza cooked in a wood oven (%)							
*Season I*	61.2	65.3	63.0	60.5	60.8	62.2	*0.888*
*Season II*	69.6*	66.9	62.6	68.1*	64.3	66.3	*0.487*

^a^significance level by chi-squared test among frequencies in the various cities

*p-value <0.05 by chi-squared test between frequencies in the two seasons

### Lifestyles and socio-economic level

The frequencies of specific children’s conditions in families with high or low socio-economic level are shown in Table 7. Exposure to passive smoking was significantly lower in families with parents’ educational level ≥26 combined years of schooling and when father had I or II occupation level as evidenced by the frequency of smoking mothers (16.2% and 16.0 respectively) and fathers (21.9 and 19.5%), presence of smokers inside the dwelling (10.8 and 9.9%) and staying of children in closed rooms with smokers (2.51 and 2.36%). Also the frequency of obese children (14.4 and 13.9%) as well as children that frequently stay in the kitchen during preparation of food (14.5 and 13.5%) is lower in families with high cultural and economic background while the residence in areas with heavy traffic is associated only with lower occupational level (40.7%). The frequency of children doing sports, including outdoor sports, is directly related to socio-economic level as opposed to frequency of children that play outdoor more than 1 h/day that it is greater in families with lower parent’s educational level (59.7%) and father’s occupational level (57.4%). Finally, children of parents with education level equal or higher than 26 combined years of schooling showed lower consumption of barbecued foods and higher consumption of toasted bread than children of parents with low educational level (<26 combined years of schooling) while children living in families where father had high level of employment (I or II level) showed lower consumption of barbecued foods, fried foods and higher of toasted bread.

**Table 7:** Association between behavioral factors and socio-economic levels of children’s families

	Parents’ overall educational level			Father’s occupational level		
	≥26 years of schooling	<26 years of schooling	*P-*value^*a*^	I or II	III or IV	*P-*value^*a*^
Mother smoker (%)	16.2	27.5	*<0.001*	16.0	24.5	*<0.001*
Father smoker (%)	21.9	43.7	*<0.001*	19.5	40.9	*<0.001*
Presence of smokers inside the dwelling (%)	10.8	20.7	*<0.001*	9.8	19.3	*<0.001*
Staying in closed rooms with smokers (%)	2.51	5.04	*0.003*	2.36	4.50	*0.006*
Obese children (%)	14.4	18.5	*0.017*	13.9	18.0	*0.009*
Frequent presence of the child in the kitchen during preparation of food (%)	14.5	19.0	*0.0105*	13.5	19.4	*<0.001*
Residence in areas with heavy traffic (%)	36.3	38.5	*0.338*	34.5	40.7	*0.003*
Sport (≥3 times/week) (%)	50.0	35.9	*<0.001*	49.2	41.4	*<0.001*
Outdoor sports (%)	35.0	25.5	*<0.001*	34.8	28.7	*0.003*
Swimming (indoor) (%)	6.42	7.15	*0.594*	5.76	8.00	*0.044*
Outdoor play (>1 h/day) (%)	48.7	59.7	*<0.0001*	48.1	57.4	*<0.001*
Consumption^b^ barbecued foods (%)	32.8	39.3	*0.005*	30.8	40.5	*<0.001*
Consumption^b^ of foods cooked on the griddle (%)	60.7	62.4	*0.482*	60.4	62.2	*0.407*
Consumption^b^ of fried foods (%)	77.4	77.5	*0.998*	75.8	80.0	*0.021*
Consumption^b^ of toasted bread (%)	53.4	46.3	*0.003*	54.0	47.5	*0.003*
Consumption^b^ of smoked foods (%)	17.2	15.3	*0.320*	16.5	17.0	*0.766*
Consumption^b^ of pizza cooked in a wood oven (%)	65.1	61.8	*0.149*	65.4	62.4	*0.151*

^a^significance level by chi-squared test

^b^in the month before the survey

## Discussion

Data on children’s recruitment and questionnaire administration activities allowed to establish the level of participation to the study. Initially, 1767 parents (56.2% of the 3144 invited) agreed to participate in the study. Although the various Research Units used the same recruitment protocol, the response rate varied across the cities involved, with the highest percentage of consenting parents in BS (67.8%) and the lowest in LE (45.7%). The reasons for this difference probably lie in the greater sensitivity to problems linked to atmospheric pollution (and the consequently greater awareness of the health risks) among citizens resident in areas in which pollution is tangible than persons who live in areas where pollution is less evident [27].

In addition, participation in our study was lower than other studies [28, 29] conducted on Italian children attending primary school in order to assess body size as a function of family, environmental and behavioral factors. In these studies the field activities were very discreet, being limited to recording the children’s anthropometric data and distributing a questionnaire to the parents. In contrast, in the MAPEC study, the length of the questionnaire and the sampling of oral mucosa cells and saliva in order to detect early genotoxic effects in recruited children-despite being carried out with non-invasive methods that were readily tolerated by the children-probably induced many parents to not participate.

The exclusion criteria and the requirement for follow-up further reduced the number of children to 1,164. The composition of the final cohort was homogeneous in terms of gender, age and nation of birth. However, a selection bias could have been generated as detectable by the parents’ level of education and rate of employment which appear slightly higher in our study than in general Italian population [28]. On the other hand, the percentage of foreign parents appears to be comparable, and the differences detected among the various cities seem to reflect data on the percentage of foreign citizens in the respective populations [16].

The BMI relative to study cohort also appear to be comparable with data from other studies [30] and confirm a higher proportion of overweight children in the central-southern regions, as better described in a specific publication on the association between weight status and lifestyles among the children enrolled in MAPEC_LIFE study [31]. This could be correlated also with poor eating habits and specifically with a low adherence to the Mediterranean diet especially in the cities of southern Italy [32].

Regarding the state of health and respiratory diseases in particular, a greater prevalence of bronchitis with production of catarrh was observed in the northern cities (BS and TO), probably associated with the colder climate or the greater levels of environmental pollution that have been widely documented in the Pianura Padana [19, 21, 22]. In this regard, several studies have shown an increase in many respiratory disorders, comprising persistent phlegm, in children in the more polluted areas [33–36]. The climates of the cities may also have influenced the frequency of physical exercise, with reference to open-air activities, which are less frequent in winter and in the colder cities.

Regarding the analysis of exposure factors linked to the home context, it emerged that the proportion of children living in areas with high traffic intensity is greatest in TO. However, the perceived intensity of traffic depends on both subjective factors and the specific areas where the children participating in the study live, which may not be representative of the city as a whole.

In contrast, the choice of fuel used for heating depends heavily on the environmental policies adopted in the various cities and on the utilities available to citizens heating their homes. Thus, in BS and TO, district heating, with “remote” production of heat, is the norm. This system serves to reduce the use of polluting fuels in urban areas with a high level of air pollution. In contrast, in LE, PG and PI, natural gas is preferred, and is also the main fuel used for cooking in all the cities participating in the study. Among the other types of heating, the use of wood-fired stoves is most frequent in PG while open-hearth fires are most common in LE in winter and in TO in spring, probably following the switch-off of the centralized or remote systems while cold temperatures continue.

Exposure to passive smoking affects large part of the cohort: one child in three among those participating in the study has at least one smoking parent and one child in seven lives together with persons who smoke inside home although only 3.2% of the interviewees admitted the presence of their offspring together with a person who smokes in closed rooms. Moreover, data analysis reveals that the fathers were more likely to be smokers, and smoking parents were most frequent in TO.

On average one child in six among those participating in the study is usually present in the kitchen during the preparation of foods and is thus potentially exposed to genotoxic compounds such as acrylamide, heterocyclic amines, nitrosamines and polyaromatic hydrocarbons, which are formed during cooking and are released into air indoors in vapor and smoke [37]. Exposure to these contaminants was also significant in terms of the ingestion of foods subject to “risky” cooking methods (barbecued, griddled, fried, toasted, smoked) [38, 39]. In some cases there were significant differences between the two seasons or between the various cities, probably due to the different regional gastronomic traditions or the different size of the foreign component and consequently the different influence of “exotic” diets with respect to autochthonous food habits.

Data on the association between lifestyle and socio-cultural level of the children’s families show that education and employment of the parents strongly affect children’s exposure to toxic contaminants in the home environment as passive smoking, cooking fumes and compounds originating from vehicular traffic as well as obesity, physical activity, ingestion of toxic substances arising from risky cooking methods. It is known that high occupational and educational levels entail access to goods and services (e.g., gymnasia and sports facilities, wellness centers, quality foods and restaurants, domestic help, etc.), which can create a protective environment in terms of various health problems [40–42]. In addition, dieting and health-promoting behaviors such as reducing smoking and high energy food and fat intake, a high level of exercise are more common in families of a higher socio-economic status [43, 44].

In summary, analysis of the data arising from the questionnaire filled in by the parents of the children participating in the MAPEC_LIFE study provides a sufficiently detailed picture of the composition of the cohort, the habits of the children and their potential exposure to certain environmental pollutants linked to the domestic context. Differences emerged with regard to the sampling period and the geographical areas. The latter could depend on different environmental, cultural and social aspects of the various cities involved in the study such as foreign citizens’ rate, economic level of the population, local food habits, climatic aspects. These findings need to be taken into account when relating the frequency of biomarkers in the children’s oral mucosa cells with the air concentration of genotoxic substances near the schools attended by the enrolled subjects.

Finally, it is important to point out that many of the children participating in the study were found to be exposed to pollution factors often attributable to ill-advised behaviors on the part of the parents. This highlights the need to perform at various levels appropriate communication interventions concerning human health risks from air pollution and the measures to prevent them. As demonstrated in a pilot study within the MAPEC_LIFE project in order to evaluate the effectiveness of some educational tools regarding pollution [45], children are very sensitive to environmental issues and demonstrate to learn very quickly the concepts about the correct lifestyles to prevent adverse health effects from exposure to harmful substances. However, as pointed out in other studies [46], it is necessary above all to teach parents and the community about the environmental hazards for human health, including those linked to lifestyles, educating them about the special vulnerability of children and showing them how to protect their children by adopting practices that reduce the risks of exposure. Previous studies indicated that active communication with the pediatricians [47], the information through mass-media [46] and the involvement of parents in school education programs [48] could contribute to improve the perception of the risk associated with exposure to contaminants in the domestic environment and to adopt health-promoting behaviors. Improvement of the parent-child communication processes may also reduce individual risk factors [49].

### Limitations

This study is not without its limitations. First, participation to the study was modest probably due to the length of the questionnaire or to biomonitoring analysis performed on recruited children. This may have generated an a priori selection bias, detectable by factors linked to the family context. The anthropometric data used in this work to calculate BMI were self-reported by the parents who compiled the questionnaires and were not measured in accordance with standardized methods. In addition, other factors (e.g., traffic near home or school, presence of the children in closed rooms with smokers) could be affected by subjective perception.

## Conclusions

This study allowed to obtain information about exposure factors linked to the home context and lifestyle of 1,164 children aged 6–8 years attending primary schools in five Italian cities with varying geographical, environmental, demographic and socio-economic characteristics.

Frequency of some factors appeared different in terms of the survey season (physical activity in the open air, the ways of cooking certain foods) and among the various cities (parents’ level of education and rate of employment, sport, traffic near the home, type of heating, exposure to passive smoking, ways of cooking certain foods). The socio-economic level seems to affect the lifestyles of children enrolled in the study including those that could cause health effects. Many factors are linked to the geographical area and may depend on environmental, cultural and social aspects of the city of residence.

Information on outdoor and indoor environmental exposure and the lifestyles of participating children will be integrated with the results of environmental and biological monitoring and with other information acquired during the MAPEC_LIFE study in order to construct a global model of genotoxic risk that can be used to support environmental policies.

## Abbreviations

BMI: Body mass index
BS: Brescia
IOTF: International Obesity Task Force
LE: Lecce
MAPEC: Monitoring Air Pollution Effects on Children for Supporting Public Health Policy
MN: Micronucleus
NW: Normal weight
OB: Obese
OW: Overweight
PG: Perugia
PI: Pisa
PM: Particulate matter
TO: Torino
UW: Underweight
